# Comparative Molecular and Genetic Landscape of Gonadal Germ Cell Tumors: Insights from Testicular and Ovarian Neoplasms

**DOI:** 10.3390/cancers18101554

**Published:** 2026-05-11

**Authors:** Diana Elena Parteni, Mihaela Camelia Tîrnovanu, Elena Țarcă, Alina Belu, Alina Jehac, Ludmila Lozneanu, Simona Eliza Giusca, Carmen Ungureanu, Eugenia Morosan, Elena Cojocaru

**Affiliations:** Grigore T. Popa University of Medicine and Pharmacy Iasi, 700115 Iasi, Romania; parteni.diana-elena@d.umfiasi.ro (D.E.P.); mihaela.tirnovanu@umfiasi.ro (M.C.T.); belu_alina@d.umfiasi.ro (A.B.); alina.jehac@umfiasi.ro (A.J.); ludmila.lozneanu@umfiasi.ro (L.L.); simona-eliza.giusca@umfiasi.ro (S.E.G.); carmen.ungureanu@umfiasi.ro (C.U.); eugenia.morosan@umfiasi.ro (E.M.); elena2.cojocaru@umfiasi.ro (E.C.)

**Keywords:** gonadal germ cell tumors, testicular germ cell tumors, ovarian germ cell tumors, cytogenetics, epigenetics, somatic mutations, tumor classification, biomarkers, germ cell neoplasia in situ, isochromosome 12p

## Abstract

**Background/Objectives**: Gonadal germ cell tumors (GCTs) arise in the testis and ovary from primordial germ cells. Despite shared origins, they display distinct molecular and genetic features. Understanding these differences is essential for clarifying tumor pathogenesis and improving diagnostic and therapeutic strategies. This review aims to compare these features and to better define the biological differences between testicular and ovarian GCTs. **Methods**: We reviewed cytogenetic alterations, epigenetic modifications, and somatic mutations reported in testicular and ovarian GCTs. Comparative analysis was performed to identify common and site-specific mechanisms. **Results**: Isochromosome 12p is a hallmark of testicular GCTs, while ovarian GCTs show diverse chromosomal changes. DNA methylation and other epigenetic marks differ by tumor subtype and gonadal origin. Somatic mutations affect pathways regulating cell cycle, pluripotency, and differentiation, with overlapping and unique patterns between testicular and ovarian tumors. Collectively, these findings define a molecular framework that explains both shared biology and gonadal-specific divergence in GCTs. **Conclusions**: Understanding molecular similarities and differences across gonadal GCTs can refine classification, guide biomarker discovery, and inform translational research. This comparative perspective highlights core pathogenetic mechanisms and gonadal-specific features relevant to clinical and experimental oncology.

## Simple Summary

Gonadal germ cell tumors (GCTs) arise from primordial germ cells in the testis and ovary, exhibiting a spectrum of histological subtypes and molecular features. While they share core pathogenetic mechanisms, testicular and ovarian GCTs differ in incidence, cytogenetic alterations, epigenetic patterns, and somatic mutations. This review integrates current evidence from clinical studies, molecular profiling, and preclinical models to provide a comparative perspective on gonadal GCTs. Understanding both shared and site-specific molecular characteristics has implications for tumor classification, biomarker development, and translational research. Highlighting these differences may guide future diagnostic and therapeutic strategies.

## 1. Introduction

Gonadal germ cell tumors (GCTs) comprise a heterogeneous group of neoplasms derived from primordial germ cells and encompass a wide spectrum of histological subtypes and biological behaviors [1,2]. According to the World Health Organization classification of Paediatric Tumours (5th edition), these tumors arise predominantly in the testis and ovary and include both benign and malignant entities with distinct developmental trajectories and clinical outcomes [3]. Testicular GCTs are the most common solid tumors in young adult males, whereas ovarian GCTs are rare but often exhibit aggressive behavior, particularly in pediatric and adolescent populations [4,5,6]. Although GCTs share overlapping histological subtypes in males and females, they differ substantially with respect to incidence and age at presentation [3].

Advances in molecular genetics and epigenomics have revealed that GCTs, despite their histological similarities, harbor distinct cytogenetic alterations, epigenetic profiles, and somatic mutations according to gonadal origin. Isochromosome 12p, for example, is a hallmark of testicular GCTs, whereas ovarian GCTs display a more heterogeneous pattern of chromosomal changes. Understanding these molecular and genetic differences is essential for refining tumor classification, identifying robust biomarkers, and informing translational research and therapeutic strategies [2,3].

This review provides a narrative synthesis of GCTs, integrating insights from developmental biology, tumor heterogeneity, and molecular genetics with current clinical and translational knowledge. A structured literature search was performed in PubMed/MEDLINE, Scopus, and Web of Science, with emphasis on studies published between 2016 and 2026, while earlier seminal publications were retained when relevant for classification or key biological concepts. Priority was given to classification documents, large clinicopathological series, molecular and genomic studies, and recent reviews that directly informed the comparison between testicular and ovarian germ cell tumors.

Because ovarian germ cell tumors are less extensively characterized than testicular germ cell tumors, particularly at the molecular level, comparative statements were interpreted carefully, especially when based on small cohorts, mixed-site studies, or extrapolation from testicular-dominant datasets.

## 2. Epidemiology and Subtype Distribution of Gonadal GCTs

GCTs occur in both sexes across a wide age range, with distinct incidence patterns depending on histological subtype. Overall, these tumors show a characteristic concentration in adolescents and young adults [1]. In the ovary, mature teratoma represents the most frequent benign GCT, whereas the corresponding testicular prepubertal-type teratoma is rare. Among malignant subtypes, seminoma (testis) and dysgerminoma (ovary) are predominant, although peak incidence occurs approximately ten years earlier in females than in males. Other malignant subtypes, including yolk sac tumor, embryonal carcinoma, and mixed GCTs, show sex- and age-specific distributions [3].

Furthermore, in males, the classification of teratoma and yolk sac tumor is strongly influenced by prepubertal versus postpubertal status, a distinction that is not recognized in females. Testicular type II GCTs arise from germ cell neoplasia in situ (GCNIS), which can remain latent until postpubertal hormonal activation and acquisition of genomic alterations such as isochromosome 12p [2,3,4]. This prolonged preclinical phase may contribute to the later clinical presentation of seminoma in young adult males. In contrast, a direct GCNIS counterpart has not been definitively established in the ovary, and dysgerminoma appears to arise within a distinct ovarian developmental and microenvironmental context [5,6]. Additionally, the association between dysgerminoma and gonadal dysgenesis or differences of sex development may further contribute to its earlier age distribution in females [7,8].

Collectively, these clinical and epidemiological differences support a cautious comparative approach. Although testicular and ovarian GCTs share several histological and developmental features, their biological interpretation is shaped by distinct gonadal microenvironments, hormonal contexts, developmental timing, and age-related classification frameworks. To facilitate interpretation, Table 1 compares the main testicular and ovarian GCT subtypes according to age distribution, relative proportion, serum markers, and immunohistochemical profile. Because published frequencies and age distributions vary across pediatric and adult cohorts, study design, and histological definitions, the values are presented as approximate ranges or qualitative descriptors rather than fixed estimates.

## 3. Histopathological Spectrum

The defining features of each germ cell tumor subtype are well established and largely overlapping between the sexes. A key distinction, however, is the presence of GCNIS in testicular GCTs, a recognized precursor lesion that has no definitive ovarian counterpart [3].

This difference also illustrates the need to distinguish between age-based and biology-based classification frameworks. In this review, the terms prepubertal/pediatric and postpubertal/adult are used mainly as clinical and epidemiological categories, whereas type I and type II GCTs refer to developmental and biological categories defined by differences in presumed cell of origin, precursor lesions, genomic alterations, and tumor behavior. These systems frequently overlap, but they are not interchangeable. Age-based terminology is therefore used when describing clinical distribution and presentation, while type I/type II terminology is applied when discussing developmental origin, precursor lesions, cytogenetic patterns, and molecular pathogenesis [3,6].

According to the WHO classification, certain histological entities are also sex-specific or predominantly associated with a single gonad. These include spermatocytic tumor, which occurs exclusively in the testis, and ovarian-specific or ovarian-characteristic lesions such as monodermal teratomas and specialized teratomas, including struma ovarii and carcinoid tumors arising within teratomas [3]. Given these biological and classification-related differences, the present review adopts a structured comparative approach. Each subsection has therefore been organized to include testicular GCTs, ovarian GCTs, and a dedicated comparative synthesis, allowing the similarities and differences between the two groups to be highlighted in a systematic manner.

### 3.1. Embryogenesis of Gonadal GCTs

GCTs reflect the intrinsic properties of primordial germinal cells (PGCs), which migrate and colonize the genital ridges independently of genetic sex. Consequently, these tumors retain remarkably similar morphological and molecular profiles in both the testis and ovary. PGCs can be identified in the wall of the yolk sac as early as weeks 3–4 post-conception, prior to gonadal formation. They subsequently undergo a complex migratory process toward the genital ridge, where definitive gonadal development occurs. Depending on somatic gonadal signaling, PGCs then commit to one of two major differentiation pathways: spermatogenesis or oogenesis [32,33].

PGC migration is regulated by multiple guidance cues, including chemokines, environmental factors, and interactions with the extracellular matrix. Genetic or epigenetic dysregulation at any stage of this process—PGC specification, migration, or gonadal colonization—may predispose to tumorigenesis, supporting the concept of GCTs as developmental disorders rather than conventional malignancies [34,35,36].

Consistent with the embryologic migration pathway of PGCs, GCTs may also arise outside the gonads in extragonadal locations. These tumors are characteristically distributed along the midline of the body, including the mediastinum, retroperitoneum, pineal and suprasellar regions, and the sacrococcygeal area [37,38]. This distinctive anatomical pattern is thought to reflect the persistence or aberrant arrest of migrating PGCs along their developmental route from the yolk sac endoderm to the genital ridges. Such ectopic germ cells may retain pluripotent potential and, under permissive conditions, give rise to extragonadal GCTs [39,40].

Following gonadal colonization, germ cell behavior diverges according to the local microenvironment. The somatic, hormonal, and stromal context of the gonad may determine whether germ cells remain quiescent, proceed toward normal gametogenesis, or undergo malignant transformation, as illustrated by the increased germ cell tumor risk observed in disorders of sex development characterized by abnormal gonadal differentiation [7,8,36]. This microenvironmental influence provides a plausible explanation for why, despite a shared embryological origin, the spectrum and relative frequency of GCTs subtypes differ between the testis and ovary.

Signaling pathways that regulate PGCs survival, migration, and proliferation during embryonic development are increasingly implicated in germ cell tumorigenesis. Among these, the PI3K/PTEN/AKT pathway plays a conserved role in germ cell development and gonadal function. Dysregulation of this signaling axis may promote abnormal cell survival and proliferation, providing a mechanistic link between early germ cell biology and the molecular events underlying the initiation of GCTs [41]. The PI3K/PTEN/AKT axis may also influence the epigenetic and transcriptional landscape of GCTs by modulating downstream regulators involved in chromatin accessibility, cellular plasticity, and metabolic adaptation. Through mTOR signaling, FOXO transcription factors, GSK3β/β-catenin interactions, and MYC-associated transcriptional programs, pathway activation can affect cell-cycle control, apoptosis resistance, and stemness-related transcriptional states. At the molecular level, activation of PI3K/PTEN/AKT signaling may converge on downstream targets involved in proliferation, survival, and metabolism, including cyclin D1, p27/Kip1, BAD/BCL-2-related signaling, GLUT1, and mTOR-dependent anabolic programs [41]. Therefore, PI3K/PTEN/AKT dysregulation should be interpreted not only as a survival pathway, but also as a potential modulator of developmental and epigenetic programs in a tumor- and gonad-specific context [41].

The biological consequences of PI3K/PTEN/AKT dysregulation may differ between testicular and ovarian GCTs. In testicular tumors, pathway activity should be interpreted in relation to germ cell neoplasia in situ, pubertal endocrine changes, and the transition from developmental arrest to invasive growth. In ovarian GCTs, the same axis may be influenced by ovarian stromal signals, follicular dynamics, and the developmental stage of the transformed germ cell [39,40,41].

**Figure 1:**
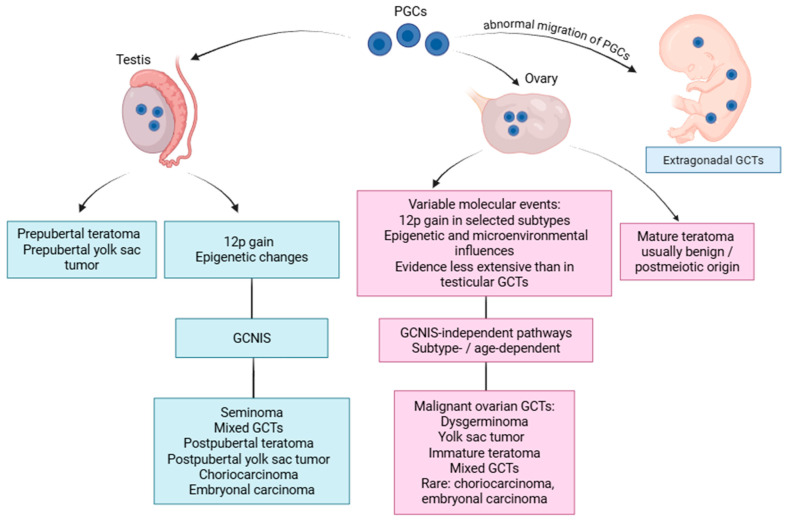
Conceptual model of divergent developmental and molecular pathways in testicular and ovarian gonadal germ cell tumors. Created in BioRender.com. The figure illustrates established and proposed pathways linking primordial germ cells to testicular, ovarian, and extragonadal GCTs. The testicular pathway distinguishes prepubertal tumors from postpubertal GCNIS-related tumors. The ovarian pathway is shown as GCNIS-independent, but should be interpreted as heterogeneous and non-linear, because molecular events such as chromosome 12p gain, epigenetic changes, and microenvironmental influences vary across subtypes and remain less extensively validated than in testicular GCTs. Therefore, the ovarian branch is intended as a conceptual synthesis rather than a definitive developmental sequence.

The developmental pathways of gonadal GCTs, including primordial germ cell migration, gonadal differentiation, and divergence into testicular and ovarian tumorigenesis, are summarized in Figure 1.

### 3.2. Molecular Pathogenesis and Developmental Subtypes

The contemporary understanding of GCT pathogenesis is largely based on the developmental model proposed by Looijenga and Oosterhuis, which stratifies germ cell tumors according to patient age, the developmental stage of the cell of origin, and distinct genetic and cytogenetic features [2]. This framework has subsequently been incorporated into the WHO Classification of Tumours and remains widely used in both clinical and molecular studies [3,4,6,42].

Within this model, type I and type II GCTs represent broad biology-based categories that frequently correspond to prepubertal and postpubertal presentation, respectively, particularly in testicular tumors. However, these terms should not be used as direct substitutes for age-based categories, and their application to ovarian GCTs requires caution because ovarian tumors are GCNIS-independent and biologically heterogeneous. Type I GCTs are classically associated with early-life presentation and are represented mainly by teratomas and yolk sac tumors, although age at diagnosis and developmental category should not be considered fully interchangeable. These neoplasms are not associated with GCNIS and typically lack chromosome 12p alterations. In contrast, type II GCTs are usually linked to postpubertal tumorigenesis and young adult presentation, especially in the testis. This group includes seminoma and non-seminomatous components such as embryonal carcinoma, postpubertal-type yolk sac tumor, choriocarcinoma, and postpubertal-type teratoma. In the testis, these tumors generally arise from GCNIS and are molecularly characterized by gain of the short arm of chromosome 12, frequently as isochromosome i(12p) [3]. This chromosomal gain increases the copy number and expression of several genes located on 12p, including CCND2 and NANOG. CCND2 promotes cell-cycle progression and proliferation [43], whereas NANOG is a key regulator of pluripotency and stem-cell maintenance [44]. Their combined overexpression may contribute to the proliferative capacity and stem-like phenotype characteristic of postpubertal-type GCTs [43,45,46].

Ovarian GCTs should be interpreted within this framework with caution. Although they share several developmental and molecular features with testicular GCTs, they are not preceded by GCNIS and remain biologically heterogeneous. Therefore, ovarian GCTs should be viewed less as direct counterparts of testicular GCTs and more as related tumors in which developmental timing, histological subtype, and the gonadal microenvironment may modify the underlying biology.

Recent integrated genomic analyses further support this developmental dichotomy. In a comprehensive study of pediatric and adolescent GCTs, Xu et al. demonstrated that type I tumors are frequently associated with chromosomal losses involving 1p and 6q, whereas type II tumors consistently exhibit 12p gain. Importantly, this cytogenetic framework applies to both testicular and ovarian germ cell tumors, despite differences in age distribution and histological subtype prevalence between the two gonads [42].

### 3.3. Cytogenetic Profile

#### 3.3.1. Testicular GCTs Cytogenetics

Testicular GCTs are characterized by marked aneuploidy and extensive large-scale chromosomal gains and losses. Integrated genomic analyses, including whole-exome/whole-genome sequencing (WES/WGS) and copy-number profiling, have identified recurrent gains involving chromosomes 2p, 7, 8, 12, 14q, 15q, 17q, 21q, and X, as well as frequent losses of Y, 4, 5, 11q, 13q, and 18q, with reported frequencies approximately in the range of 25–40% across different cohorts [47].

The cytogenetic hallmark of type II testicular GCTs is gain of the short arm of chromosome 12, most commonly in the form of isochromosome 12p (i(12p)), which is detected in more than 80% of postpubertal tumors, including both seminomatous and non-seminomatous subtypes. Classical and modern cytogenetic approaches (FISH, SNP array, mate-pair sequencing) indicate that 12p overrepresentation is associated with tumor invasion and progression from GCNIS but does not represent a very early event: GCNIS lesions may lack i(12p), whereas invasive tumors almost invariably harbor this alteration [3,4].

In addition, type II testicular GCTs display a distinct ploidy pattern, with seminomas generally being hypertriploid and non-seminomatous tumors tending to be hypotriploid [3,4]. In contrast, prepubertal testicular teratomas and yolk sac tumors (type I) are typically near-diploid and lack i(12p), further supporting their pathogenetic separation from GCNIS-derived neoplasms [2,4,42].

#### 3.3.2. Ovarian GCTs Cytogenetics

Cytogenetic data specific to ovarian GCTs are relatively limited due to their rarity; however, early comparative genomic hybridization (CGH) analyses and more recent genomic profiling studies have begun to clarify their chromosomal alteration landscape. In a CGH study of 21 malignant ovarian GCTs (including dysgerminomas, yolk sac tumors, and mixed tumors), recurrent copy-number gains were observed at 12p, 21, 8, and 1q, while the most common deletion involved chromosome 13. This profile indicates that malignant ovarian GCTs share genomic features with testicular GCTs, though immature teratomas exhibited few consistent gains or losses, suggesting distinct underlying biology [48].

Data regarding DNA ploidy in ovarian germ cell tumors are limited but suggest heterogeneous patterns. Flow-cytometric studies have demonstrated that the majority of ovarian dysgerminomas exhibit nondiploid DNA content, reflecting underlying chromosomal instability [49]. Analyses of malignant ovarian GCTs have shown that aneuploid or tetraploid tumors tend to display more complex genomic alterations, supporting the association between ploidy abnormalities and chromosomal instability [50,51].

More recent genomic analyses using whole-exome sequencing and copy-number profiling in a cohort of 87 ovarian GCTs confirmed that, overall, these tumors have low somatic mutation rates, with frequent 12p gains present across most histological subtypes, except in pure immature teratomas. In addition to chromosome 12p copy-number gain, focal amplifications involving oncogenic pathways such as PI3K/AKT/PTEN have been identified, as well as recurrent focal deletions affecting multiple chromosomal regions (1p36.32, 2q11.1, 4q28.1, 5p15.33, 5q11.1, and 6q27) [52]. Among the recurrent chromosomal deletions reported in ovarian GCTs, loss of the 1p36 region is of particular interest. Deletions affecting this locus have been described in germ cell tumors, particularly in yolk sac tumors, suggesting the presence of tumor-suppressor genes involved in germ cell tumorigenesis [53,54]. More broadly, the 1p36 region has been recognized as a tumor-suppressor locus in several malignancies, and its loss has been associated with aggressive tumor behavior [55].

Together, these data indicate that the cytogenetics of ovarian GCTs is characterized by recurrent gains of chromosome 12p and other chromosomal regions, a relatively low mutation burden, and structural abnormalities that may overlap with, but also diverge from those observed in testicular GCTs, particularly when comparing subtypes such as dysgerminoma, yolk sac tumor, and immature teratoma [6].

#### 3.3.3. Comparative Cytogenetics of Testicular GCTs and Ovarian GCTs

Despite their shared embryological origin, direct cytogenetic comparison between testicular and ovarian GCTs remains challenging because of differences in cohort size, histological composition, age distribution, and methodological approaches. Nevertheless, several recurrent chromosomal patterns support a partially shared oncogenic framework, although the strength of evidence differs substantially between the two groups.

In postpubertal testicular GCTs, gain of chromosome 12p, most commonly as isochromosome 12p or regional 12p amplification, represents a well-established cytogenetic hallmark with diagnostic and biological relevance. Its presence is closely linked to the invasive postpubertal testicular GCT pathway and supports the concept that 12p gain contributes to progression beyond early germ cell transformation. By contrast, ovarian GCTs show a more heterogeneous and less consistently defined cytogenetic profile, with abnormalities varying according to histological subtype, patient age, and developmental stage. Therefore, differences between testicular and ovarian GCTs should not be interpreted solely as the presence or absence of individual chromosomal alterations, but also in terms of their frequency, timing during tumor evolution, and biological significance within each gonadal context [56].

Overall, both testicular and ovarian malignant GCTs are characterized predominantly by extensive chromosomal imbalances rather than recurrent point mutations. Although chromosome 12p gain is most consistent in postpubertal testicular GCTs, it has also been reported in subsets of ovarian malignant GCTs, supporting partially overlapping pathogenetic mechanisms linked to germ cell tumor development [4,47,52].

In testicular GCTs, 12p gain, frequently in the form of isochromosome i(12p), is strongly associated with invasive growth and progression from GCNIS [4]. By contrast, ovarian GCTs display greater cytogenetic heterogeneity, and the significance of 12p alterations appears to vary according to histological subtype, age group, and the strength of available evidence. For example, immature teratomas may show minimal recurrent copy number alterations, supporting a developmental and biological trajectory distinct from that of postpubertal testicular GCTs [52]. Thus, cytogenetic similarities with testicular GCTs, particularly in seminoma/dysgerminoma-related patterns, should not obscure the broader heterogeneity of ovarian tumors, including yolk sac tumor, immature teratoma, mixed GCTs, and rare embryonal carcinoma or choriocarcinoma. Overall, subtype-specific interpretation is more appropriate than direct transfer of the postpubertal testicular GCT model to all ovarian GCTs [52].

A major biological distinction between testicular and ovarian germ cell tumorigenesis is the presence of a recognized pre-invasive precursor lesion in the testis but not in the ovary. In testicular GCTs, progression from GCNIS to invasive disease is closely linked to additional genomic events, particularly 12p gain [4]. By contrast, in the ovary, no equivalent “ovarian GCNIS” has been definitively established in the usual gonadal setting [6]. This suggests that ovarian germ cell tumorigenesis may follow a different developmental trajectory, potentially lacking a prolonged morphologically identifiable in situ phase and instead progressing more directly to invasive disease. Such divergence may reflect differences in germ cell maturation and in the gonadal microenvironment, although the precise mechanisms remain incompletely understood [8,56].

Importantly, comparative interpretation remains limited by the relative scarcity of large, ovary-specific genomic datasets and by historical differences in cytogenetic methodologies. Future integrative analyses using harmonized platforms across gonadal sites will be required to refine these comparisons.

### 3.4. Epigenetic Landscape

#### 3.4.1. Testicular GCTs Epigenetics

Testicular GCTs display a distinctive epigenetic landscape that reflects their primordial germ cell origin and contributes to their characteristic clinical behavior, including both high sensitivity to cisplatin-based chemotherapy in most patients and the emergence of epigenetically mediated resistance in selected cases. In contrast to many adult solid tumors, testicular GCTs are defined by a relatively low somatic mutation burden but prominent epigenetic dysregulation, which plays a central role in tumor initiation, differentiation, and therapeutic response [57,58,59].

DNA methylation represents one of the most extensively studied epigenetic mechanisms in testicular GCTs. Global methylation patterns correlate closely with histological subtype and differentiation state: seminoma-like tumors typically exhibit a globally hypomethylated profile consistent with a less differentiated germ cell phenotype, whereas non-seminomatous components—particularly those showing somatic differentiation—display progressively higher levels of DNA methylation [58,60]. These methylation differences mirror developmental programs and support the concept that epigenetic remodeling accompanies lineage commitment in testicular GCTs.

A substantial body of evidence links epigenetic alterations to cisplatin sensitivity and resistance in testicular GCTs. Although most of them are highly chemosensitive, resistant tumors often acquire a more hypermethylated epigenetic profile, a phenomenon often described as epigenetic “hardening” [47,57,59]. This shift may reflect both treatment-induced selection and differentiation-associated reprogramming, providing a possible mechanistic basis for the emergence of refractory disease and supporting interest in epigenetic therapies as chemosensitizing strategies [57,61].

Beyond DNA methylation, chromatin organization, histone post-translational modifications, and non-coding RNAs also contribute to testicular GCT biology. These mechanisms influence pluripotency networks, lineage commitment, DNA damage responses, and treatment outcome [57,58,59,62]. MicroRNA clusters miR-371~373 and miR-302/367 are consistently overexpressed across malignant germ cell tumors, with broad conservation across age groups and gonadal sites, and are considered core developmental outputs of germ cell malignancy [63,64,65]. These miRNAs have demonstrated strong clinical potential as circulating biomarkers for diagnosis, disease monitoring, and detection of residual disease.

Long non-coding RNAs have also emerged as regulators of testicular GCT biology. For example, LINC00467 and LINC00470 have been associated with proliferation, invasion, chemoresistance, and immune microenvironment changes, partly through PI3K/AKT/mTOR and TGF-β-related signaling [66,67]. In addition, experimental models suggest that differentiation-associated developmental programs, including SOX17-related WNT, TGF-β/Activin, and FGF signaling, may promote transition of embryonal carcinoma cells toward yolk sac tumor lineage, with loss of pluripotency and acquisition of cisplatin resistance [68,69].

Collectively, these findings support the relevance of epigenetic regulation in testicular GCT pathogenesis, differentiation, treatment response, and resistance, with important implications for biomarker development and therapeutic innovation.

#### 3.4.2. Ovarian GCTs Epigenetics

The epigenetic profile of ovarian GCTs is less clearly defined than that of testicular GCTs, partly because these tumors are rare and histologically diverse. For this reason, ovarian-specific findings should be distinguished from mechanisms inferred from testicular-dominant or mixed-site studies, and limited evidence should be interpreted as emerging rather than definitive.

Available data suggest two clinically relevant epigenetic observations. First, malignant ovarian GCTs may share germ cell-program miRNA signatures described across malignant GCTs, including the miR-371~373 and miR-302/367 clusters [63,65]. However, most clinical validations of circulating miRNA biomarkers, particularly for diagnosis, monitoring, and relapse detection, have been performed in testicular GCTs [70]. Therefore, their diagnostic and prognostic value in ovarian tumors remains less firmly established.

Second, ovarian teratomas show distinctive imprinting and methylation patterns. Mature ovarian teratomas show methylation profiles consistent with a maternal-only genome, concordant with a parthenogenetic origin [70]. Similarly, analysis of immature ovarian teratomas indicates broadly maternal imprinting patterns, while also suggesting that selected loci may show aberrant methylation relative to mature teratomas [71]. These findings support a subtype-specific epigenetic background in ovarian teratomas, although their broader relevance across non-teratomatous ovarian GCTs remains uncertain.

A major limitation in the study of ovarian GCTs is the scarcity of large ovary-specific methylome datasets across malignant subtypes. Nevertheless, pediatric and mixed-site GCT methylation studies provide useful comparative context, suggesting that germinoma/seminoma/dysgerminoma-type tumors tend to show lower methylation than yolk sac tumors, consistent with differences in differentiation state [72].

#### 3.4.3. Comparative Epigenetics of Testicular GCTs and Ovarian GCTs

Despite their shared origin from primordial germ cells, testicular and GCTs exhibit both overlapping and distinct epigenetic features that reflect differences in developmental timing, gonadal microenvironment, and differentiation potential. Direct epigenetic comparison is limited by the unequal availability of epigenomic datasets, with testicular tumors being substantially better characterized than ovarian counterparts [6,57,58].

A shared epigenetic hallmark of malignant GCTs is the preservation of germ cell-associated regulatory programs, particularly reflected by overexpression of the miR-371~373 and miR-302/367 clusters [63,64,65]. Among these, miR-371a-3p has attracted particular attention as a circulating biomarker. Serum or plasma miR-371a-3p can be detected using quantitative PCR-based assays and has shown high diagnostic performance in malignant GCTs, with reported sensitivities approximately in the range of 90–95% and specificities exceeding 90%, outperforming traditional markers such as AFP and β-hCG in many testicular GCT cohorts [24,73]. Circulating levels correlate with tumor burden and decline after tumor resection or chemotherapy, supporting its use in diagnosis, treatment monitoring, and relapse detection [74]. However, clinical validation remains strongest in testicular GCTs, while ovary-specific evidence is still limited. In addition, mature teratoma remains a major limitation, as it is generally not detected by miR-371a-3p-based assays [73,75].

Although miR-371a-3p is one of the best validated circulating biomarkers in testicular GCTs, particularly for diagnosis, treatment monitoring, tumor burden assessment, and relapse detection, its clinical application in ovarian GCTs remains less established. In ovarian tumors, current data support its biological relevance within the broader malignant germ cell tumor miRNA program, but further ovary-specific validation is required before it can be considered an established diagnostic or surveillance biomarker [73].

Long non-coding RNAs have been more extensively investigated in testicular than in ovarian GCTs. In testicular GCTs, XIST represents one of the earliest and most frequently discussed examples, particularly in relation to supernumerary X chromosomes, XIST promoter demethylation, and seminomatous/non-seminomatous tumor biology [76]. Other lncRNAs have been reported in association with specific tumor phenotypes or biological behavior. MEG3 has been linked to regulation of testicular GCT growth through the PTEN/PI3K/AKT axis, while TTTY14 has been proposed as a Y-linked testis-associated lncRNA associated with proliferation, prognosis, and the tumor immune microenvironment [63,64,65,77,78].

Expression-profiling studies have also identified lncRNA signatures reported to distinguish seminoma from yolk sac tumor, including XIST, C17orf86/SNHG20, FAM182A, FLJ11235, and C12orf47, as well as age-related differences involving PART1, MEG3, TP53TG1, POM121L9P, and TTTY15 [76]. However, many of these candidates remain insufficiently functionally validated and should be interpreted as emerging biomarkers rather than established oncogenic drivers.

In ovarian GCTs, the available evidence regarding lncRNAs is considerably more limited. Although ovarian germ cell tumors share developmental and epigenetic features with testicular GCTs, direct extrapolation from the testicular setting is not appropriate. Current data do not yet support a well-defined lncRNA landscape for ovarian dysgerminoma, yolk sac tumor, immature teratoma, or mixed ovarian GCTs in the same manner as described for testicular GCTs [79]. Therefore, lncRNAs in ovarian GCTs should be discussed mainly as an emerging area of investigation, with a need for tumor-specific transcriptomic studies that distinguish ovarian GCTs from epithelial ovarian cancers and from testicular GCT models [80].

Beyond non-coding RNAs, DNA methylation patterns also reveal important gonad- and subtype-specific differences. In testicular GCTs, global methylation levels correlate with histological subtype and treatment response, with seminoma-like tumors displaying relative hypomethylation and non-seminomatous or cisplatin-resistant tumors exhibiting progressive hypermethylation [47,57,58,59,60]. In ovarian GCTs, epigenetic profiling is dominated by subtype-specific features, particularly in teratomas, which show imprinting and methylation patterns consistent with a parthenogenetic origin [70,71]. Comparable data directly associating epigenetic states with chemotherapy response in ovarian germ cell tumors remain limited, reflecting both smaller cohort sizes and fewer integrative epigenomic studies rather than definitive biological absence [6,72].

Collectively, available evidence indicates that testicular and ovarian GCTs share a conserved epigenetic framework reflective of their primordial germ cell origin, while diverging in gonad-specific epigenetic programs related to imprinting, differentiation, and therapy response. These similarities and differences underscore both the biological unity and contextual divergence of the epigenetics of gonadal GCTs.

### 3.5. Mutational Profiles

#### 3.5.1. Testicular GCTs Mutations

Testicular GCTs are characterized by a relatively low burden of recurrent somatic single-nucleotide mutations compared with many other adult solid tumors, but they still harbor distinct and biologically relevant mutational events. Comprehensive analyses have identified activating mutations in KIT, KRAS, and NRAS as the most observed somatic point mutations in testicular GCTs, particularly in seminoma subtypes. KIT mutations are frequent in seminomas but less common in non-seminomatous germ cell tumors, supporting their role in driving proliferative signaling via MAPK pathways in this context [81,82].

Recent reviews highlight the central involvement of receptor tyrosine kinase signaling and downstream RAS/MAPK cascades in testicular GCTs biology. Despite the overall low mutational burden, alterations affecting KIT and RAS signaling pathways regulate key cellular processes including proliferation, differentiation, apoptosis, and stress responses. These pathways are also intimately linked to epigenetic regulation and primordial germ cell development, providing a mechanistic bridge between embryological origin and oncogenic transformation in testicular GCTs [83].

Although recurrent point mutations are uncommon in testicular GCTs, selected oncogenic alterations appear to be associated with aggressive clinical behavior. KRAS mutations have been identified in a subset of tumors, particularly in mixed histology, and are more frequently detected in metastatic disease compared with primary tumors. These observations support a role for RAS pathway activation in tumor progression and suggest that, despite their low overall frequency, such mutations may have prognostic and therapeutic relevance [84].

In a recent genomic panel study of testicular germ cell tumors, multiple somatic variants were detected across canonical oncogenes and tumor suppressors, with TP53 among the most recurrently mutated genes in an unselected cohort. This study found variants not only in KIT and KRAS but also in PDGFRA, EGFR, BRAF, NRAS, PIK3CA, MET, and ERBB2, suggesting potential actionability in select cases. Moreover, copy number alterations—particularly KRAS copy number gain—were highly prevalent (≈80%) and associated with worse prognosis, underlining the importance of structural genomic events alongside somatic point mutations in testicular GCTs pathogenesis [85].

Although somatic mutations contribute to tumor biology, testicular GCTs typically combine these events with marked chromosomal abnormalities (e.g., isochromosome 12p), and single-gene mutations remain relatively uncommon overall [86,87]. However, certain mutations, despite their low frequency, may carry important clinical implications. Notably, mutations in TP53, while infrequent in treatment-naive testicular GCTs, have been reported in cisplatin-resistant and refractory tumors [82]. This observation is particularly relevant because the exceptional cisplatin sensitivity of testicular GCTs has been partly attributed to an intact p53-mediated DNA damage response, which promotes rapid apoptosis following chemotherapy-induced DNA damage [87,88]. Alterations affecting this pathway, including TP53 mutations, have been observed in cisplatin-resistant tumors, although they account for only a subset of refractory cases [82].

#### 3.5.2. Ovarian GCTs Mutations

In ovarian GCTs, many mutational findings are based on small cohorts or selected tumor subtypes, making it important to distinguish recurrent and biologically meaningful alterations from isolated observations that still require confirmation. Whole-exome sequencing studies demonstrate a very low overall mutation rate (~0.05 mutations per megabase) in ovarian GCTs specimens compared with other cancer types, consistent with a biology dominated by chromosomal aberrations rather than recurrent point mutations [52].

Although few highly recurrent somatic mutations have been validated across large ovarian GCTs cohorts, subtype-specific trends have emerged. For instance, PI3K/AKT/PTEN pathway alterations have been suggested in yolk sac tumor components, implying that aberrant signaling within growth and survival pathways could play a role in specific histologies [52]. Some exome-based studies have also reported potential somatic mutations in MAPK1 in rare mixed germ cell tumor cases, indicating that somatic events may vary by subtype and warrant further investigation [89].

It remains notable that ovarian GCTs genomes are predominantly characterized by copy number changes and ploidy variations rather than a high frequency of recurrent somatic point mutations [52]. Putative somatic events identified in smaller cohorts include mutations in genes such as POU5F1, which potentially reflect deregulated pluripotency networks, although the clinical and mechanistic relevance of these findings requires validation in larger series [90].

#### 3.5.3. Comparative Mutational Profiles of Testicular GCTs and Ovarian GCTs

Mutational findings in GCTs should be interpreted according to their recurrence, biological plausibility, and level of clinical validation. Recurrent alterations may point to relevant driver pathways, whereas rare mutations reported in small cohorts or selected histological subtypes should be considered exploratory. Similarly, potentially actionable events remain investigational unless supported by stronger molecular and clinical evidence. This distinction is especially important in ovarian GCTs, where sequencing data are still limited and subtype-specific conclusions remain difficult.

Comparative genomic assessments illustrate that both testicular and ovarian GCTs exhibit a low somatic mutational burden relative to most carcinomas, reinforcing the concept that germ cell tumors primarily rely on structural genomic alterations and embryonic regulatory networks for oncogenesis [52,91].

A key distinction is the pattern and recurrence of point mutations: testicular GCTs, particularly seminoma, consistently display activating mutations in KIT and RAS pathway genes, whereas ovarian GCTs lack a similarly frequent and consistent set of somatic point mutations. This suggests fundamental differences in the molecular drivers—or at least in the detectable somatic events—of these tumors, possibly reflecting divergent developmental pathways and tissue-specific contexts [81,82].

Another notable comparative aspect is the role of somatic TP53 alterations. In testicular GCTs, mutations in TP53 are rare in treatment-naive tumors but emerge as a hallmark of resistance to platinum-based therapy, implicating TP53 pathway disruption as a mechanism of adaptive chemoresistance [82]. In ovarian GCTs, data on TP53 as a recurrent somatic event are limited, likely due to the lack of large-scale sequencing cohorts, although rare events cannot be excluded.

Overall, these comparative patterns emphasize that testicular GCTs and ovarian GCTs share a low baseline mutation rate but diverge in the specific somatic events that recur and shape their biology, with testicular GCTs showing more defined hotspots (KIT/RAS) and ovarian GCTs reflecting greater heterogeneity and lower recurrence of somatic mutations [52].

Table 2 summarizes the main molecular, cytogenetic, epigenetic, developmental, and microenvironmental differences and overlaps between testicular and ovarian germ cell tumors, based on currently available literature.

## 4. Integrative Insights and Future Directions

Although ovarian and testicular germ cell tumors arise from a common primordial germ cell origin, this review underscores how gonadal context critically shapes their molecular and biological divergence. Developmental timing, hormonal signaling, and somatic support within the gonad appear to be decisive factors influencing whether germ cells complete normal differentiation or remain in an embryonic-like state with malignant potential [5].

At the cytogenetic level, both tumor types share hallmark features such as chromosomal aneuploidy and recurrent gains of chromosome 12p in postpubertal tumors, supporting a unified developmental framework for gonadal germ cell tumorigenesis [4,5,6]. However, differences in the prevalence, timing, and biological consequences of these alterations suggest that gonad-specific factors modulate tumor evolution, particularly in ovarian germ cell tumors, where cytogenetic patterns appear more heterogeneous and less extensively characterized [8,52].

Epigenetic regulation represents a central unifying theme across gonadal GCTs. Global DNA hypomethylation, characteristic microRNA expression profiles, and the preservation of pluripotency-associated epigenetic programs are shared features in both ovarian and testicular tumors. Nevertheless, emerging evidence indicates that gonad-specific epigenetic trajectories, including differences in imprinting status and differentiation potential, may contribute to variability in tumor behavior and therapeutic response [6].

Clinical data from patients with disorders of sex development and dysgenetic gonads provide additional biological support for the importance of the gonadal environment in germ cell tumorigenesis. In these settings, impaired somatic support and disrupted differentiation are associated with increased tumor risk and the persistence of precursor lesions such as GCNIS or gonadoblastoma [7,92].

From a therapeutic perspective, the shared developmental and epigenetic features of germ cell tumors may partly explain their remarkable sensitivity to platinum-based chemotherapy, which remains one of the most successful examples of curative systemic therapy in solid tumors. However, a subset of tumors develops resistance or relapse, suggesting that additional molecular and microenvironmental determinants contribute to treatment response [93,94]. Improved characterization of these mechanisms may ultimately facilitate more accurate risk stratification and the development of novel therapeutic strategies for patients with refractory disease.

In addition to intrinsic genetic and epigenetic alterations, the tumor microenvironment has recently emerged as a potential contributor to germ cell tumor biology. Seminomas and dysgerminomas frequently exhibit dense lymphocytic infiltration and expression of immune checkpoint molecules such as PD-L1, reflecting an immunologically active tumor milieu [95,96,97]. Despite this immunogenic profile, clinical studies evaluating immune checkpoint inhibitors such as pembrolizumab have shown only modest activity in heavily pretreated germ cell tumors, suggesting that immune regulatory pathways are complex and incompletely understood in this disease context [98,99,100].

Future research integrating developmental biology with high-resolution genomic, epigenomic, and single-cell profiling across both gonadal contexts will be essential to clarify the mechanisms driving tumor initiation, progression, and therapeutic response. Comparative studies of ovarian and testicular germ cell tumors may therefore provide a unique model for understanding how shared developmental origins give rise to distinct tumor phenotypes and clinical outcomes. Ultimately, such integrative approaches may facilitate improved risk stratification and support the development of more precise and biologically informed therapeutic strategies.

## 5. Limitations and Strength of Evidence

A direct comparison between testicular and ovarian germ cell tumors is informative, but it is affected by an important asymmetry in the available literature. Testicular tumors have been more extensively profiled at the cytogenetic, molecular, epigenetic, and biomarker levels, whereas ovarian germ cell tumors remain less represented in large-scale studies. As a result, some parallels between the two gonadal sites are well supported, while others should be regarded as provisional. In addition, ovarian germ cell tumor studies often include small cohorts, mixed histological subtypes, or heterogeneous methodological approaches, which limits the strength of subtype-specific conclusions. Throughout this review, we therefore aimed to distinguish established shared mechanisms from observations that are still emerging or largely inferred from testicular-dominant datasets.

## 6. Comparative Synthesis: Key Similarities and Differences Between Testicular and Ovarian GCTs

Testicular and ovarian germ cell tumors are related by their germ cell origin and, in selected subtypes, by overlapping diagnostic profiles. Markers such as SALL4, OCT3/4, PLAP, CD117, AFP, and hCG remain useful in both locations, although their expression must always be interpreted in relation to tumor subtype and clinical context. Beyond these shared features, important differences separate the two groups. Postpubertal testicular GCTs follow a relatively well-defined developmental model, strongly associated with GCNIS and frequent chromosome 12p gain, most often represented by i(12p). Ovarian GCTs, by contrast, do not fit as easily into this model. They are more heterogeneous, lack a clearly established GCNIS-equivalent precursor, and show molecular changes that vary according to histological subtype and age.

Thus, the comparison between testicular and ovarian GCTs is useful, but it should not imply biological equivalence. Testicular GCTs provide a better-characterized framework, particularly for postpubertal tumors, whereas ovarian GCTs require a more cautious, subtype-specific interpretation. The available data support areas of overlap, especially in diagnostic immunophenotype and selected molecular pathways, but also emphasize that ovarian tumors cannot simply be interpreted through the model established for testicular disease. This distinction is central for a balanced comparative approach.

## 7. Summary and Conclusions

Germinal cell tumors are a biologically unified yet clinically diverse group of neoplasms. Despite overlapping histology, testicular and ovarian GCTs arise from distinct developmental and molecular contexts, with GCNIS marking a precursor stage unique to the testis. Cytogenetic, epigenetic, and somatic mutation profiles reveal both shared tumorigenic pathways and sex-specific divergences, underpinning differences in onset, behavior, and prognosis.

Integrating epidemiological, histopathological, and molecular insights provides a framework for refined classification, biomarker development, and precision therapeutics, emphasizing the need to consider both unity and divergence in future research and clinical management.

## Figures and Tables

**Table 1:** Comparative overview of age distribution, relative frequency, serum biomarkers, and immunohistochemical profiles, and subtype-specific comparative notes in testicular and ovarian gonadal germ cell tumors.

Subtype	Testis: Age	Testis: Proportion	Ovary: Age	Ovary: Proportion	Serum Markers	IHC Markers	Comparative Note
Benign teratoma	Prepubertal-type teratoma, typically <6 years [9,10]	~40% of intratesticular tumors in young children [10,11]	Mature teratoma; peak in reproductive age [12,13]	~69% of ovarian GCTs [14]	Usually normal [15]	Usually negative for OCT3/4 and SALL4, unless undifferentiated malignant germ cell components are present [16]	Pediatric predominance in testis contrasts with reproductive-age predominance in ovary.
Malignant teratoma	Postpubertal-type teratoma; median age ~29 years [17]	~3% of testicular GCTs [17]	Immature teratoma; adolescents and young adults [18,19]	~33–55% of malignant ovarian GCTs [18,19]	Usually normal; AFP may be mildly increased in immature or mixed tumors [20]	Variable SALL4, especially in immature neuroepithelial or primitive elements [20]	Testicular postpubertal teratoma and ovarian immature teratoma are not biologically equivalent.
Seminoma/dysgerminoma	Peak incidence 25–34 years [21,22]	~58–82% of testicular GCTs [21,22]	Adolescence to young adulthood, mainly teens and twenties [19,23]	~29–38% of malignant ovarian GCTs [19,23]	LDH may be increased; hCG may be variably increased [15,24]	SALL4, OCT3/4, CD117, and D2-40 positive [20,25]	Closest diagnostic parallel between testicular and ovarian GCTs.
Yolk sac tumor/endodermal sinus tumor	Predominantly prepubertal boys, usually <6 years; pure postpubertal tumors are uncommon and often mixed [26]	Pure form <5% of testicular GCTs [22]	Median age ~18 years [25]	~15% of malignant ovarian GCTs [19]	AFP increased [15]	AFP, SALL4, and glypican-3 positive [20]	Similar marker profile, but different age distribution between sites.
Mixed germ cell tumor	Young adults [27]	~45% of testicular GCTs [27]	Mainly adolescents and young adults; mean age ~15 years in some series [19,28]	~8–17% of malignant ovarian GCTs [19,28]	Depends on histological components	Depends on histological components	Requires component-based interpretation in both sites.
Choriocarcinoma, non-gestational	Young adults [29,30]	<5% of testicular GCTs [22]	Children, adolescents, and young adults [19]	~1% of malignant ovarian GCTs [19]	hCG increased [24]	hCG positive; glypican-3 may be variable [20]	Rare in both sites, but particularly uncommon in the ovary.
Embryonal carcinoma	Young adults [31]	~16% of testicular GCTs [31]	Adolescents and young adults [25]	Extremely rare [25]	AFP and/or hCG may be increased, especially in mixed tumors [15]	CD30, SALL4, and OCT3/4 positive [20]	Recognized in testicular GCTs, but exceptional as a primary ovarian tumor.

Note: Reported age ranges and subtype frequencies are approximate and should be interpreted in relation to cohort composition, pediatric versus adult inclusion criteria, referral patterns, histological definitions, and study design. Serum and immunohistochemical markers reflect commonly reported diagnostic profiles, but may vary according to tumor purity, mixed histology, differentiation status, and clinical context. For ovarian germ cell tumors, which are less represented in large molecular and clinicopathological series, broader ranges or qualitative descriptors were used when precise numerical estimates could be misleading.

**Table 2:** Comparative overview of testicular and ovarian germ cell tumors.

Feature	Testicular GCTs	Ovarian GCTs	Comparative Interpretation
Cytogenetics	frequent 12p gain/i(12p)	less consistent, subtype-dependent	shared germ cell origin, but different frequency and biological weight
Epigenetics	GCNIS-related patterns, imprinting abnormalities	subtype- and developmental-stage dependent	divergent timing of germ cell maturation may influence tumorigenesis
Mutational burden	generally low/moderate, subtype-dependent	variable, often subtype-specific	both are not classically mutation-driven tumors
Microenvironment	testicular niche, pubertal/hormonal context	ovarian stromal and follicular context	gonadal microenvironment may modulate progression

## Data Availability

No new data were created or analyzed in this study. Data sharing is not applicable to this article.

## Abbreviations

AFP: Alpha-fetoprotein; AKT: Protein kinase B; CCND2: Cyclin D2; CD30: Cluster of differentiation 30; CD117: Cluster of differentiation 117; CGH: Comparative genomic hybridization; D2-40: Podoplanin; DNA: Deoxyribonucleic acid; GCNIS: Germ cell neoplasia in situ; GCTs: Germ cell tumors; hCG: Human chorionic gonadotropin; i(12p): Isochromosome 12p; KIT: KIT proto-oncogene receptor tyrosine kinase; KRAS: Kirsten rat sarcoma viral oncogene homolog; LDH: Lactate dehydrogenase; lncRNAs: Long non-coding RNAs; MAPK: Mitogen-activated protein kinase; miRNAs: MicroRNAs; mTOR: Mechanistic target of rapamycin; NANOG: Nanog homeobox; OCT3/4: Octamer-binding transcription factor 3/4; PGCs: Primordial germ cells; PI3K: Phosphoinositide 3-kinase; PTEN: Phosphatase and tensin homolog; RAS: Rat sarcoma viral oncogene homolog; RNA: Ribonucleic acid; SALL4: Spalt-like transcription factor 4; TGF-β: Transforming growth factor beta; WES: Whole-exome sequencing; WGS: Whole-genome sequencing; WHO: World Health Organization; WNT: Wingless/integrated.
